# Different lipid profiles, insulin sensitivity, and insulin resistance among Han, Uygur, and Kazak men with normal glucose tolerance in Xinjiang, China

**DOI:** 10.1186/s12944-018-0863-9

**Published:** 2018-09-07

**Authors:** Yan Wang, Jun Zhang, Yanrong Ma, Xiangxin Song, Suli Li, Xianqin Zhan, Lan Wu

**Affiliations:** 1 Endocrinology Department of Xinjiang Uygur Autonomous Region People’s Hospital, Urumqi, Xinjiang, 830001 China; 2 School of Medicine, Shihezi University, Shihezi, Xinjiang, 832000 China; 3 Xinjiang Medicine University, Urumqi, Xinjiang, 830001 China

**Keywords:** Lipid metabolism, Glucose metabolism, Insulin sensitivity, Insulin resistance, Epidemiology, Ethnic differences, Han, Uygur, Kazak

## Abstract

**Background:**

This study aimed to determine the differences in clinical parameters among Han, Uygur, and Kazak men with normal glucose tolerance.

**Methods:**

Participants’ data from the China National Diabetes and Metabolic Disorders Study pertaining to Han, Uygur, and Kazak men from the Xinjiang province were used (*n* = 930). Pearson’s correlation was used to examine the relationship between HOMA-IR, Matsuda Index, and clinical characteristics.

**Results:**

HOMA-IR of Han men was significantly higher than in Uygurs and Kazaks (*P* < 0.001). The Matsuda Index of Kazaks was significantly higher than that of Hans and Uygurs (*P* < 0.001). While Kazaks had the highest BMI, WC, SBP, and DBP; they also had the highest HDL-C and lowest TG (*P* < 0.001). TG of Uygurs was significantly higher than that of Hans and Kazaks (P < 0.001). In Hans and Kazaks, the TG/HDL-C ratio increased with HOMA-IR quartiles; there was no association in Uygurs. In Hans and Kazaks, the TG/HDL-C ratio decreased with Matsuda index quartiles; there was no association in Uygurs. Multivariate linear regression showed that HOMA-IR was independently associated with ethnicity, BMI and TG/HDL-C ratio (*P* < 0.01), while Matsuda index was independently associated with ethnicity, BMI, LDL-C levels (*P* < 0.001) and TG/HDL-C ratio (P < 0.001).

**Conclusions:**

In conclusion, Han, Uygur, and Kazak men had different lipid profiles, BMI, and WC. Han men had the highest insulin resistance while Kazak men had the highest insulin sensitivity.

## Background

Diabetes and prediabetes are major public health issues in China. According to the studies by the China National Diabetes and Metabolic Disorders Study Group (CNDMDS) performed from 2007 to 2008 in 46,239 adults from 14 provinces in China, the prevalence of diabetes in China was 9.7% [1]. Furthermore, the prevalence of prediabetes in men was 15.5% and 16.6% in urban and rural areas, respectively [2]. Finding and controlling risk factors such as lipid profile disorders before the occurrence of diabetes are critical to control this epidemic.

In previous studies by our group and others, we observed that the lipid profiles of Han, Uygur, and Kazak people with prediabetes were very different [3–6]. Interestingly, Kazaks were found to have a lower prevalence of diabetes compared with Uygurs, while Hans had the highest risk of diabetes even after adjustment for body mass index (BMI) or waist circumference (WC). Kazaks were more prone to hypertension than the other two populations [7].

The index of insulin resistance in homeostasis model assessment (HOMA-IR) [8] and the Matsuda index [9] are good predictors of diabetes mellitus development even in individuals with normal glucose tolerance [10]. The HOMA-IR reflects hepatic insulin sensitivity and basal hepatic glucose production [8], while the Matsuda Index combines both hepatic and peripheral tissue insulin sensitivity [9]. Furthermore, among the lipid parameters, the triglycerides (TG) to high-density lipoprotein cholesterol (HDL-C) ratio is associated with HOMA-IR and Matsuda index, and is also an independent predictor of cardiometabolic events [11, 12]. This ratio is easier to measure in a day-to-day clinical setting than HOMA-IR and the Matsuda index. Since tools exist to detect insulin resistance in people with normal glucose tolerance, the early recognition of the disorder is very important in order to take early lifestyle and/or medical actions and limit the risk of diabetic complications.

Nevertheless, the differences in lipid profiles before prediabetes of those three populations are not known exactly. Therefore, the purpose of this study was to determine the differences in clinical parameters among Han, Uygur, and Kazak men with normal glucose tolerance, and to examine the relationship between HOMA-IR and the Matsuda index with clinical characteristics in these three populations. Men were studied because they have a higher cardiovascular risk than women and because the menopausal status is a confounding factor [13]. The present study is the first to observe and compare the lipid profiles in the Han, Uygur, and Kazak populations with normal glucose tolerance in Xinjiang, China.

## Methods

### Study population

The present study is a subset study using the Xinjiang participants and data from the CNDMDS, which was a multistage, stratified sampling study conducted from 2007 to 2008 in 46,239 adults from 14 provinces in China [1]. The exact recruitment strategy has been published [1].

The present study was approved by the ethics committee each participating institutions in the Xinjiang province. Written informed consent was obtained for the main study and for eventual substudies [1]. The need for individual consent for this subset study was waived by the committee.

The 1999 World Health Organization diagnostic criteria were used to diagnose diabetes and prediabetes [14]. Subjects with fasting glucose levels ≥6.1 mmol/L (≥110 mg/dL), 2-h glucose levels ≥7.8 mmol/L (≥140 mg/L), diagnosed diabetic, or using antihypertensive, antidiabetic, or lipid-lowering drugs were excluded.

### Data collection

Study personnel was trained to measure blood pressure and obtain anthropometric measurements and blood specimens according to a standard protocol [15]. All participants of ≥20 years of age were instructed to maintain their usual physical activity and diet for at least three days before the oral glucose tolerance test. After an 8–10-h overnight fast, venous blood samples were collected in a vacuum tube containing sodium fluoride for the measurement of plasma glucose. Participants with no history of diabetes were given a standard 75-g glucose solution. Blood samples were drawn at 0, 30, and 120 min after the glucose load to measure glucose and insulin levels. Plasma glucose levels were measured with the use of a hexokinase enzymatic method. Serum cholesterol and triglyceride levels were assessed enzymatically using commercially available reagents.

### Definitions

The HOMA-IR was calculated as [8]:

HOMA-IR = (I_0_ × G_0_)/22.5.

where I_0_ is the fasting plasma insulin concentration (mIU/L) and G_0_ is the fasting plasma glucose concentration (mmol/L).

The Matsuda index was calculated as [9]:

$$ =\frac{10000}{\sqrt{\mathbf{G0}\times \mathbf{10}\times \mathbf{Gmean}\times \mathbf{Imean}}} $$

Matsuda Index.

where I_0_ is the fasting plasma insulin concentration (mIU/L), G_0_ is the fasting plasma glucose concentration (mg/dl), G_mean_ is the mean plasma glucose concentration during OGTT (mIU/l), and I_mean_ is the mean plasma insulin concentration during OGTT test (mg/dl).

The TG/HDL-C ratio was calculated as TG (mmol/L) divided by HDL-C ratio (mmol/L).

### Statistical analysis

No sample size was performed since it a cohort substudy that included all available patients who met the eligibility criteria. All patients are from the original CNDMDS cohort [1]. The clinical characteristics of the study population were analyzed using one-way ANOVA with the LSD post hoc test. The relationships between HOMA-IR and Matsuda Index with the clinical characteristics were examined using Pearson’s correlations. In order to normalize their distribution, log_2_ HOMA-IR and log_2_ Matsuda index were used. TG/HDL-C ratio, Log_2_ HOMA-IR, and Log_2_ Matsuda index were stratified into quartiles. The four quartiles of Log_2_ HOMA-IR were (in Hans, Uygurs, and Kazaks, respectively): Q1: < 0.385, < 0.137, and < 0.123; Q2: 0.385–0.632, 0.137–0.434, and 0.123–0.185; Q3: 0.632–0.946, 0.434–0.756, and 0.185–0.518; and Q4: > 0.946, > 0.756, > 0.518. The four quartiles of Log_2_ Matsuda index were (in Hans, Uygurs, and Kazaks, respectively): Q1: < 1.533, < 1.921, and < 2.163; Q2: 1.533–1.892, 1.921–2.263, and 2.163–2.484; Q3: 1.892–2.293, 2.263–2.564, and 2.484–2.776; and Q4: > 2.293, > 2.564, and > 2.776. Multiple linear regression was performed (forward method; candidate variables were age, gender, BMI, WC, SBP, DBP, and lipid profile; non-normally distributed variables were ln transformed) to identify the factors independently associated with HOMA-IR and the Matsuda index. All analyses were conducted using SPSS 19.0 for Windows (IBM, Armonk, NY, USA). Two-sided *P*-values < 0.05 were considered statistically significant.

## Results

### Clinical characteristics of the Han, Uygur, and Kazak men

The characteristics of the participants are shown in Table 1. All subjects had normal serum glucose values. HOMA-IR of Han men was significantly higher than in Uygurs and Kazaks (2.08 vs. 1.82 vs. 1.40, *P* < 0.001; Han > Uygur>Kazak), while the Matsuda Index of Kazaks was significantly higher than that of Hans and Uygurs (7.67 vs. 10.25 vs. 13.04, P < 0.001; Kazak>Uygur>Han).

**Table 1:** Clinical characteristics of Han, Uygur, and Kazak men from the Xinjiang province (*n* = 930)

Mean (95% CI)	Han (*n* = 325)	Uygur (*n* = 423)	Kazak (*n* = 182)
Age (years)	41.2 (39.7–42.6)	44.0 (42.7–45.3)	45.5 (43.5–47.5)
BMI (kg/m^2^)	24.3 (24.0–24.7)	24.6 (24.3–24.9)	26.1 (25.5–26.7)
WC (cm)	82.7 (81.7–83.8)	85.7 (84.8–86.7)	87.9 (86.2–89.7)
SBP (mmHg)	116 (114–118)	113 (111–115)	132 (129–135)
DBP (mmHg)	77 (76–78)	75 (73–76)	86 (84–88)
Serum glucose (mmol/l)			
Fasting	5.03 (4.97–5.09)	5.36 (5.32–5.40)	4.52 (4.47–4.58)
30 min	8.26 (8.05–8.47)	7.50 (7.33–7.67)	7.22 (6.99–7.46)
120 min	5.41 (5.30–5.53)	5.21 (5.11–5.31)	5.14 (5.03–5.26)
Serum insulin (U/l)			
Fasting	9.32 (8.87–9.77)	7.34 (6.98–7.69)	6.97 (6.42–7.53)
30 min	60.97 (54.55–67.40)	35.48 (32.69–38.28)	27.42 (23.66–31.18)
120 min	30.20 (26.89–33.50)	17.79 (16.17–19.41)	15.29 (13.49–17.09)
TC (mmol/l)	4.58 (4.47–4.69)	4.98 (4.89–5.08)	5.09 (4.96–5.23)
TG (mmol/l)	1.47 (1.34–1.60)	2.01 (1.95–2.08)	0.92 (0.83–1.01)
HDL-C (mmol/l)	1.34 (1.31–1.37)	1.16 (1.13–1.19)	1.71 (1.69–1.74)
TG/HDL-C ratio	1.14(1.05–1.02)	1.82(1.75–1.90)	0.54 (0.49–0.59)
HOMA-IR	2.08 (1.98–2.18)	1.74 (1.66–1.83)	1.40 (1.29–1.51)
Matsuda index	7.67 (7.21–8.12)	10.25 (9.82–10.68)	13.04 (12.17–13.91)

BMI: body mass index; WC: waist circumference; SBP: systolic blood pressure; DBP: diastolic blood pressure; TC: total cholesterol; TG: triglycerides; HDL-C: high-density lipoprotein cholesterol; HOMA-IR: index of insulin resistance in homeostasis model assessment.

Normal values: TG < 2.26 mmol/L; TC < 6.22 mmol/L; HDL-C > 1.04 mmol/L. All subjects had normal serum glucose values.

Kazaks were significantly older than Hans (*P* < 0.01), while Uygurs were significantly older than Hans (*P* < 0.05). BMI (P < 0.01), WC (P < 0.01), and systolic and diastolic blood pressure (SBP and DBP) (*P* < 0.001) of Kazaks were significantly higher than those of Hans and Uygurs (Kazak>Uygur>Han).

While Kazaks had the highest BMI, WC, SBP, and DBP, they also had the highest HDL-C (Kazak>Han > Uygur) and lowest TG (*P* < 0.001). TG of Uygurs was significantly higher than that of Hans and Kazaks (P < 0.001; Uygur>Han > Kazak). There were no significant differences in LDL-C among the three populations.

### Correlations of HOMA-IR and Matsuda index with clinical characteristics

Interestingly, we found that the data were different among the three populations. In Hans, BMI (*r* = 0.271, *P* < 0.001), WC (*r* = 0.245, *P* < 0.001), TC (*r* = 0.129, *P* = 0.020), TG (*r* = 0.142, *P* = 0.010), HDL-C (*r* = − 0.153, *P* = 0.006), LDL-C (*r* = 0.195, *P* < 0.001), and TG/HDL-C (*r* = 0.180, *P* = 0.001) were correlated with HOMA-IR, while BMI (*r* = − 0.295, *P* < 0.001), WC (*r* = − 0.327, P < 0.001), SBP (*r* = − 0.125, *P* = 0.024), TC (*r* = − 0.199, *P* < 0.001), TG (*r* = − 0.263, P < 0.001), HDL-C (*r* = 0.304, *P* < 0.001), LDL-C (*r* = − 0.368, P < 0.001), and TG/HDL-C ratio (r = − 0.327, *P* < 0.001) were correlated with the Matsuda index (Table 2).

**Table 2:** Pearson correlations of HOMA-IR and Matsuda index with clinical characteristic in Han, Uygur, and Kazak men (n = 930)

		BMI	WC	SBP	DBP	TC	TG	HDL-C	LDL-C	TG/HDL-C ratio
Han										
HOMA-IR	PCC	0.271†	0.245†	0.089	0.064	0.129*	0.142*	− 0.153†	0.195†	0.180†
Matsuda index	PCC	−0.295†	− 0.327†	− 0.125*	− 0.105	−0.199†	− 0.263†	0.304†	− 0.368†	−0.327†
Uygur										
HOMA-IR	PCC	0.198†	0.234†	0.081	0.039	0.134†	0.055	0.066	0.111*	− 0.011
Matsuda index	PCC	−0.273†	−0.274†	− 0.088	−0.065	− 0.070	−0.065	− 0.072	−0.128†	− 0.004
Kazak										
HOMA-IR	PCC	0.161*	0.074	−0.006	0.056	0.068	0.166*	0.046	−0.031	0.165*
Matsuda index	PCC	−0.306†	−0.233†	0.045	−0.098	− 0.110	−0.260†	− 0.104	−0.023	− 0.259†

*P < 0.05

†*P* < 0.01

Adjusted for age

*BMI*: body mass index; *WC*: waist circumference; *SBP*: systolic blood pressure; *DBP*: diastolic blood pressure; *TC*: total cholesterol; *TG*: triglycerides; *HDL-C*: high-density lipoprotein cholesterol; *HOMA-IR*: index of insulin resistance in homeostasis model assessment; *PCC*: Pearson correlation coefficient

In Uygurs, BMI (*r* = 0.198, *P* < 0.001), WC (*r* = 0.234, P < 0.001), TC (*r* = 0.134, *P* = 0.006), and LDL-C (*r* = 0.111, P < 0.001) were correlated with HOMA-IR, while BMI (*r* = − 0.273, *P* < 0.001), WC (*r* = − 0.274, P < 0.001), and LDL-C (*r* = − 0.128, *P* = 0.008) were correlated with the Matsuda index (Table 2).

In Kazaks, BMI (*r* = 0.161, *P* = 0.031), TG (*r* = 0.166, *P* = 0.026), and TG/HDL-C ratio (*r* = 0.165, *P* = 0.027) were correlated with HOMA-IR, while BMI (*r* = − 0.306, *P* < 0.001), WC (*r* = − 0.223, *P* = 0.002), TG (*r* = − 0.260, P < 0.001), and TG/HDL-C ratio (*r* = − 0.259, P < 0.001) were correlated with the Matsuda index (Table 2).

In all three populations, HOMA-IR was highly correlated with the Matsuda index (*P* < 0.001).

### TG/HDL-C ratio according to quartiles of log_2_ HOMA-IR and log_2_ Matsuda index in Han, Uygur, and Kazak men

**Fig. 1:**
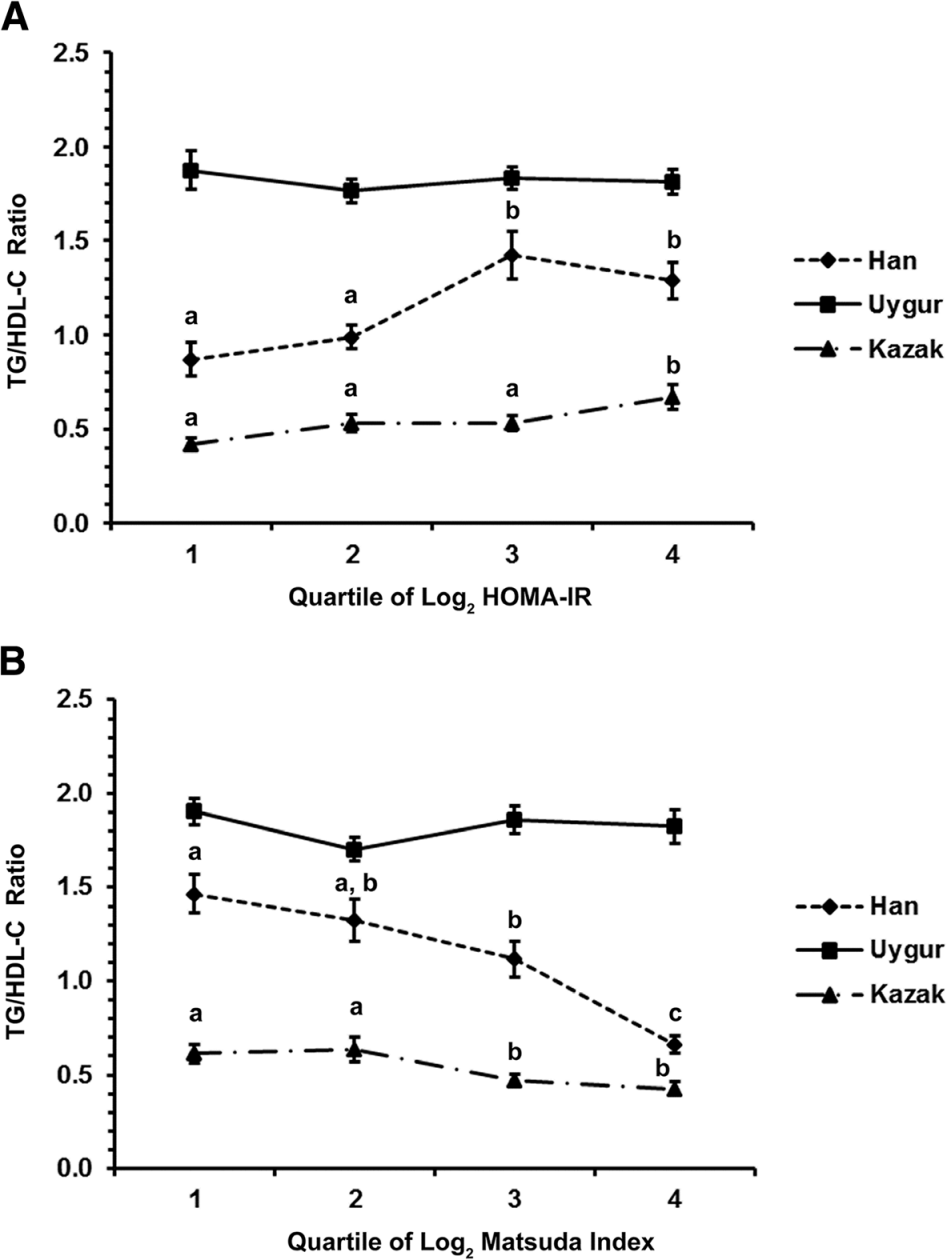
**a** TG/HDL-C ratio according to HOMA-IR among Han, Uygur, and Kazak men. **b** TG/HDL-C ratio according to the Matsuda index among Han, Uygur, and Kazak men. TG/HDL-C ratio denoted by different letters indicated significant difference between quartiles

In Hans, the TG/HDL-C ratio increased with log_2_ HOMA-IR quartiles and was the highest at Q3 (0.87 vs. 0.99 vs. 1.42 vs. 1.29, P < 0.001, *P* < 0.05 for Q1 and Q2 vs. Q3 and Q4). In Kazaks, the TG/HDL-C ratio increased with log_2_ HOMA-IR quartiles and was the highest at Q4 (0.42 vs. 0.53 vs. 0.53 vs. 0.67, *P* = 0.006, *P* < 0.05 for Q4 vs. Q1, Q2, and Q3). There was no association between TG/HDL-C and log_2_ HOMA-IR quartiles in Uygurs (*P* = 0.77) (Fig. 1).

In Hans, the TG/HDL-C ratio decreased with log_2_ Matsuda index quartiles and was the highest at Q1 (1.47 vs. 1.32 vs. 1.12 vs. 0.66, *P* < 0.001, *P* < 0.05 for Q1 vs. Q3 vs. Q4 and for Q2 vs. Q4). In Kazaks, the TG/HDL-C ratio decreased with log_2_ Matsuda index quartiles and was the highest at Q2 (0.61 vs. 0.64 vs. 0.47 vs. 0.43, *P* = 0.004, P < 0.05 for Q1 and Q2 vs. Q3 and Q4). There was no association between TG/HDL-C and log_2_ Matsuda index quartiles in Uygurs (*P* = 0.26) (Fig. 1).

### Multivariate analysis

Multivariate analyses were performed to see whether the lipid profile played a role in the difference in insulin resistance of the different ethnic groups. The multivariate analysis of HOMA-IR showed that the TG/HDL-C ratio was independently associated with HOMA-IR (*P* = 0.003) (Table 3). On the other hand, the multivariate analysis of the Matsuda index showed that the LDL-C levels (*P* < 0.001) and TG/HDL-C ratio (P < 0.001) were independently associated with the Matsuda index (Table 3).

**Table 3:** Multivariate analysis of factors associated with HOMA-IR and Matsuda index

Variables	Beta	Lower 95%CI	Upper 95%CI	*P*
HOMA-IR				
Ethnicity (Han)^a^	0.224	0.182	0.266	< 0.001
Ethnicity (Uygur) ^a^	−0.035	− 0.091	0.020	0.208
BMI	0.023	0.015	0.032	< 0.001
TG/HDL-C	0.090	0.031	0.148	0.003
Matsuda index				
Ethnicity (Han) ^a^	−0.318	−0.360	− 0.276	< 0.001
Ethnicity (Uygur) ^a^	0.099	0.043	0.154	< 0.001
Age	0.004	0.002	0.007	< 0.001
BMI	−0.034	−0.042	−0.025	< 0.001
LDL-C	−0.124	−0.173	− 0.076	< 0.001
TG/HDL-C	−0.127	− 0.189	− 0.066	< 0.001

^a^Reference = Kazak

*BMI*: body mass index; *LDL-C*: low-density lipoprotein cholesterol; *TG*: triglycerides; *HDL-C*: high-density lipoprotein cholesterol

## Discussion

The lipid profiles and diabetes risk indicators of Han, Uygur, and Kazak people with prediabetes are different [3–6], but the changes in lipid profiles before prediabetes of those three populations are unknown. Therefore, this study aimed to determine the differences in clinical parameters among Han, Uygur, and Kazak men with normal glucose tolerance. The results showed that Han, Uygur, and Kazak men had different lipid profiles, BMI, and WC. Han men had the highest insulin resistance while Kazak men had the highest insulin sensitivity among those three populations.

Many studies such as ACCORD, ADVANCE, and The Veterans Affairs Diabetes Trial [16–19] showed that intensive glucose control is beneficial to prevent the microvascular complications of diabetes, but those studies did not include macrovascular complications such as cardiovascular diseases and stroke. Nevertheless, if clinicians intervene when obesity, hypertension, dyslipidemia, and/or diabetes mellitus are diagnosed, it is already too late since some damage is already done. Therefore, the early recognition of glucose/insulin disorders is very important in order to take early lifestyle and/or medical actions and limit the risk of diabetic complications.

The present study is the first study to observe the lipid characteristics in Han, Uygur, and Kazak populations with normal glucose tolerance in Xinjiang, China. Although Kazaks had the highest BMI, WC, SBP, and DBP, they also had the highest HDL-C and lowest TG. TG levels of Uygurs were significantly higher than that of Hans and Kazaks. The exact reasons for those differences are unknown, but genetic and environmental factors (diet, stress, and physical activity) could be, at least in part, responsible for those differences. Possibly as a consequence of differences in lipid profiles, HOMA-IR of Hans was the highest, while Kazaks’ was the lowest, and an inverse relationship was observed for the Matsuda index. Those observations are in line with the prevalence of diabetes mellitus of 8.0%, 6.0%, and 3.5% in Hans, Uygurs, and Kazaks, respectively [7]. HOMA-IR [8] and the Matsuda index [9] are good predictors of diabetes mellitus development even in individuals with normal glucose tolerance [10], but it is important to highlight that they do not represent exactly the same biological processes. Indeed, the HOMA-IR reflects hepatic insulin sensitivity and basal hepatic glucose production [8], while the Matsuda Index combines both hepatic and peripheral tissue insulin sensitivity [9].

Based on a previous study by our group, the diagnosis of metabolic syndrome (MS) is based on WC cut-off of 85 cm for Hans and 90 cm for Uygurs and Kazaks [20]. In the present study, WC was not independently associated with HOMA-IR and Matsuda index, but BMI was. According to an African descent study, it was shown that even though insulin resistance, cardiovascular diseases, and type 2 diabetes are associated with hypertriglyceridemia, African descent individuals with these conditions usually have normal TG levels [1], suggesting the presence of the “TG paradox” [21]. A previous study showed that non-diabetic African-American adults had a more favorable lipid profile despite high rates of cardiovascular disease [22]. It seems that TG levels below the current MS threshold criterion are associated with insulin resistance in African-Americans [22]. As syndromes are formulated to identify individuals at high risk for conditions such as cardiovascular disease, MS, or type 2 diabetes, ethnic differences in plasma lipid levels have to be considered [23].

The present study showed that BMI, WC, HOMA-IR, and Matsuda index of Han’s are associated with the lipid profile, while those parameters are only associated with TC among Uygurs and only with TG among Kazaks. A TG/HDL-C ratio > 3.5 is a simple mean of identifying insulin resistance in dyslipidemic patients who are likely to be at increased risk of cardiovascular disease [24]. In addition, in the Korean population, high TG/HDL-C ratio is associated with insulin resistance according to WC [25]. Interestingly, in the present study, TG/HDL-C ratio was associated with HOMA-IR and the Matsuda index in Hans and Kazaks, but not in Uygurs. Based on these results, it could be hypothesized that in Hans, a TG/HDL-C ratio of 1 means a higher risk of insulin resistance. Nevertheless, those parameters are different in Uygurs and Kazaks, which is consistent with previous reports indicating that Uygurs had a significantly greater risk of diabetes than Kazak [2, 26].

In the present study, SBP and DBP were associated with BMI and WC in all three populations, but these correlations included TG, LCL-C, and TC in Hans, and only TC in Uygurs and Kazaks. According to a study by Zhang Jun et al. [27], at the same BMI, Uygurs have greater waist to hip ratio values, abdominal visceral fat content, and diabetes risk than Kazaks. These differences were mainly associated with the distribution of adipose tissue in the body, changes in metabolic activity, and adipokine secretion by the adipose tissue [27, 28]. Once again, genetics and lifestyle habits could play roles in those differences, but additional studies are necessary.

The present study is not without limitations. First, the study population was relatively small and from a single geographical area. The available data were limited to those originally collected for the CNDMDS study. The sample size was unequal among the three ethnic groups because the SNDMDS study was originally designed to represent the demographic distribution of the ethnic groups in China [1]. Finally, the main limitation of the present study is that no dietary evaluation was performed. Nevertheless, those three ethnic groups are known to have different dietary habits in terms of fat and salt consumption [29], which could have explained, at least in part, some of the results observed here. Additional studies are still necessary to comprehensively understand the differences of cardiovascular and diabetes risks in Hans, Uygurs, and Kazaks.

## Conclusions

Control of BMI and WC is vital to avoid metabolic diseases [30–33]. In conclusion, Han, Uygur, and Kazak men had different lipid profiles, BMI, and WC. Han men had the highest insulin resistance while Kazak men had the highest insulin sensitivity. Therefore, it is possible that interventions of TG/HDL-C should be the main focus in Han men, while TC control could be more important to Uygur and Kazak men. Strict blood pressure control could be a key point in Kazak men to prevent cardiovascular diseases. Future interventional studies are necessary to examine these possibilities.

## Abbreviations

BMI: Body mass index
CNDMDS: China National Diabetes and Metabolic Disorders Study Group
HDL-C: High-density lipoprotein cholesterol
HOMA-IR: Homeostasis model assessment
TG: Triglycerides
WC: Waist circumference
